# Accelerated 3YMD programs: the last decade of growth of the Consortium of Accelerated Medical Pathway Programs (CAMPP)

**DOI:** 10.1080/10872981.2024.2400394

**Published:** 2024-09-27

**Authors:** Catherine L. Coe, Sally A. Santen, Annette C. Reboli, Jeffrey R. Boscamp, Amanda M. Stoltz, Erin Latif, Lisa Grill Dodson, Matthew Hunsaker, Anuradha Paavuluri, Judith Brenner, Seethalakshmi Ramanathan, Allison Macerollo, Shou Ling Leong, Lisa Strano-Paul, Christin Traba, Betsy Goebel Jones, Kristen Rundell, Alicia Gonzalez-Flores, William J. Crump, Mark Vining, April O. Buchanan, Debaroti Tina Mullick Borschel, Christina M. Vitto, Joan Cangiarella

**Affiliations:** a Family Medicine, University of North Carolina School of Medicine, Chapel Hill, NC, USA; b Emergency Medicine, Virginia Commonwealth University School of Medicine, Richmond, VA, USA; c Emergency Medicine, University of Cincinnati College of Medicine, Cincinnati, OH, USA; d Medicine, Cooper Medical School of Rowan University, Camden, NJ, USA; e Hackensack Meridian School of Medicine, Nutley, NJ, USA; f Family Medicine, James H. Quillen College of Medicine, East Tennessee State University, Johnson City, TN, USA; g Obstetrics and Gynecology, Medical College of Georgia at Augusta University, Augusta, GA, USA; h Department of Family and Community Medicine, Medical College of Wisconsin - Central Wisconsin, Wausau, WI, USA; i Family Medicine, Medical College of Wisconsin - Green Bay, Green Bay, WI, USA; j Pediatrics, Mercer University School of Medicine, Macon, GA, USA; k Medical Education, New York University Grossman Long Island School of Medicine, Mineola, NY, USA; l Psychiatry and Behavioral Sciences, Norton College of Medicine, SUNY Upstate Medical University, Syracuse, NY, USA; m Department of Family and Community Medicine, The Ohio State University College of Medicine, Columbus, OH, USA; n Family and Community Medicine, Pennsylvania State University College of Medicine, Hershey, PA, USA; o Medicine, Department of Internal Medicine, Renaissance School of Medicine at Stony Brook University, Stony Brook, NY, USA; p Pediatrics, Rutgers New Jersey Medical School, Newark, NJ, USA; q Department of Medical Education; Texas Tech University Health Sciences Center, Lubbock, TX, USA; r Department of Family and Community Medicine, University of Arizona, College of Medicine- Tucson, Tucson, AZ, USA; s Internal medicine, University of California Davis School of Medicine, Davis, CA, USA; t Family Medicine, University of Louisville School of Medicine Trover Campus, Madisonville, KY, USA; u Pediatrics, University of Massachusetts Chan Medical School, Worcester, MA, USA; v Professor of Pediatrics, University of South Carolina School of Medicine Greenville, Greenville, SC, USA; w University of Tennessee Health Science Center, College of Medicine, Memphis, TN, USA; x Competency Based Graduation Program and Program Emergency Medicine and Internal Medicine, Virginia Commonwealth University, Richmond, VA, USA; y Pathology, New York University Grossman School of Medicine, New York, NY, USA

**Keywords:** Accelerated curriculum, medical education, workforce, three-year MD, Debt reduction

## Abstract

**Introduction:**

Over the past decade, the growth of accelerated three-year MD (3YMD) programs has flourished. In 2015, with support from the Josiah Macy Jr. Foundation, the Consortium of Medical Pathway Programs (CAMPP) started with eight North American medical schools. The objective of this paper is to evaluate the current state of the 3YMD programs.

**Material and Methods:**

Since 2015, the CAMPP has tracked new and prospective 3YMD programs. An electronic survey collecting curricular and programmatic information about the programs was disseminated to all members of the CAMPP in August 2023. The survey included elements related to year of initiation, number of graduates, and curricular elements.

**Results:**

Of the schools with known established three-year MD programs, 29 of 32 programs responded (response rate 90%). There is growth of Accelerated Medical Pathway Programs over time with almost 20% of United States Allopathic Medical Schools having or developing an accelerated program. There have been 817 graduates from these programs from 2013–2023. Most schools include an opportunity for a ‘directed pathway’ experience for students. A directed pathway is where a student completes the MD degree in three-years and then has a direct placement into an affiliated residency program, provided they meet the goals and objectives of the curriculum. Most of the schools report a mission to reduce medical student debt and build a workforce for a specialty, for a population of patients, or geographical distribution.

**Conclusions:**

Accelerated three-year medical pathway programs have grown significantly over the last decade, consistent with an overall effort to redesign medical curricula, reduce debt and contribute to the workforce.

## Introduction

In 1910, the Flexner Report outlined the structure of medical education to include two years of pre-clinical work followed by two years of clinical experiences in an apprenticeship model. The four-year framework became the standard format for the Medical Doctorate (MD) degree.

While there have been several times in history where medical schools have transitioned to a three-year MD curriculum to facilitate physician shortages, many ceased to exist due to changes in physician workforce demands and funding [1,2]. In the last 15 years, there has been a resurgence of interest in accelerated medical education programs to address physician workforce shortages, provide individualized education pathways in specific specialties, and to respond to the rising cost of medical education and subsequent student debt [3–5]. Additionally, the constant questioning of the value of the fourth-year fuels this change [6]. The Consortium of Accelerated Medical Pathway Programs (CAMPP) was established in 2015 through a grant from the Josiah Macy Jr. Foundation. Eight North American medical schools with accelerated three-year MD (3YMD) pathways joined together to form this consortium with the mission to develop a workforce to practice in underserved and rural areas and individualize students’ education to allow them to enter the specialty that interests them one year early. While the mission of the schools differs, they shared a common goal of debt reduction for the students.

The goals of the consortium were to 1) disseminate best practices in developing an accelerated pathway, 2) to offer solutions to common challenges and suggestions towards optimal resource allocation, and 3) to participate in collaborative research and disseminate the meaningful outcomes of the accelerated pathway programs through publications and presentations at national and international meetings [3]. Schools with established accelerated curricula or prospective schools are eligible for membership within CAMPP. With rekindled interest in accelerated three-year MD pathways, the consortium has quadrupled in size. In 2017, Academic Medicine published an article that described three-year MD programs with perspectives from CAMPP [3]. In this article, we address the growth of three-year MD pathways with description of the goals of each program, their admissions model, their timelines for United States Medical Licensing Exams (USMLE), and their connection with residency programs. The objective of this paper is to describe the current state of the three-year MD programs and program characteristics.

## Materials and methods

Since 2015, the CAMPP has tracked new and prospective accelerated pathway programs. An electronic survey collecting curricular and programmatic information about the programs was disseminated to all members of the CAMPP in August 2023. The survey included elements related to year of initiation, number of graduates, and curricular elements and these are reported below. The survey was completed by the 3YMD program director. This was a non-human subject program evaluation as we were collecting information about the program and not human subjects. This study was determined to not require human subjects review through the NYU Grossman School of Medicine IRB’s self-certification process in that the data were collected for program evaluation purposes and the study team had access only to aggregate, anonymous data.

## Results

Of the schools with known established 3YMD programs, 29 of 32 programs responded (response rate 90%). Program descriptions for each respondent are highlighted below. There have been 817 graduates from these programs from 2013–2023. Table 1 outlines the program information and goals of the program, including the year the program started, the connection to residency, the number of graduates, and the mission.

**Table 1.** Description of three-year accelerated programs start year, specialty focus, number of graduates and goals of the program.

School	Year of first matriculated class	Directed pathway to residency (Yes (Y) or No (N)	Specialty focus	Number of graduates as of June 2023	Goals of the Program
Cooper Medical School of Rowan University	2016	Y	Internal Medicine, Pediatrics, Primary care	12	To prepare the next generation of primary care physicians who will be providing patient-centered, humanistic, and culturally-sensitive care for patients and families in New Jersey and beyond.
Geisinger Commonwealth	2022	Y	Family Medicine, Internal Medicine	0	Expand the physician workforce in Pennsylvania. Reduce student debt.
Hackensack Meridian	2018	Y	Anesthesiology. Child Neurology, Emergency Medicine, Family Medicine, Internal Medicine, Neurology, Obstetrics and Gynecology, Pediatrics, Physical Medicine and Rehabilitation, Psychiatry, Surgery	80	Expand the physician workforce in New Jersey. Reduce student debt. Provide a seamless transition between UME and GME.
James H. Quillen College of Medicine- East Tennessee State University	2023	Y	Family Medicine, Internal Medicine, Pediatrics	0	Allow students who are certain of their career choice to graduate early. Reduce student debt.
Medical College of Georgia	2021	Y	Emergency Medicine, Family Medicine, Internal Medicine, Obstetrics and gynecology, Pediatrics, Psychiatry, Surgery	5	Expand the physician workforce in Georgia. Reduce student debt.
Medical College of Wisconsin – Central	2016	N	Primary Care, Psychiatry	62	Community based primary care and psychiatry focused to address physician workforce shortages in Wisconsin and surrounding areas. Reduce student debt.
Medical College of Wisconsin– Green Bay	2015	N	Primary Care, Psychiatry	140	Community based primary care and psychiatry focused to address physician workforce shortages in Wisconsin and surrounding areas. Reduce student debt.
Medical University of South Carolina	2017	Y	Emergency Medicine, Family Medicine, Internal Medicine, Neurology, Obstetrics and gynecology, Pathology, Pediatrics, Psychiatry	7	Expand the physician workforce in South Carolina. Reduce student debt.
Mercer University School of Medicine	2012	Y	Family Medicine, Internal Medicine, Pediatrics	65	Address Georgia’s critical shortage of primary care physicians who practice in medically underserved rural areas. Reduce student debt.
New York University Grossman	2013	Y	Anesthesiology, Cardiothoracic Surgery, Dermatology, Emergency Medicine, Internal Medicine, Neurology, Neurosurgery, Obstetrics and Gynecology, Orthopedic Surgery, Ophthalmology, Otolaryngology, Pathology, Pediatrics, Plastic Surgery, Primary Care, Psychiatry, Radiation Oncology, Radiology, Rehabilitation Medicine, Surgery, Urology	158	Individualize medical education to allow those who are certain of their career choice to graduate early. Track learners across the UME-GME continuum. Reduce student debt.
New York University Grossman Long Island	2019	Y	Internal medicine, Obstetrics and gynecology, Pediatrics, General Surgery	45	Increase the workforce in primary care to serve the needs of the Long Island New York community. Train physicians in health system science. Reduce student debt.
Norton College of Medicine, SUNY Upstate Medical University	2023	Y	Emergency Medicine, Internal Medicine, Neurology, Pediatrics, Psychiatry, Radiation Oncology, Surgery	0	Optimize and individualize medical school training. Retain physicians in the Upstate NY region. Streamline and improve UME-GME transition. Reduce student debt
Ohio State University College of Medicine	2017	Y	Family Medicine with goals to increase to other Primary Care Specialties	10	Graduating more Ohio primary care physicians faster and addressing national and Ohio shortages of primary care physicians. Reduce student debt.
Penn State College of Medicine	2014	Y	Emergency Medicine, Family Medicine, Internal Medicine, Neurology, Orthopedics, Pathology, Psychiatry, Radiology, Urology	30	Individualize and accelerate medical education. Increase workforce, especially in primary care and train patient-centered physicians. Reduce student debt.
Renaissance School of Medicine Stony Brook University	2018	Y	Anesthesiology, Dermatology, Emergency Medicine, Family Medicine, Internal Medicine, Medicine-Pediatrics, Neurology, Obstetrics and Gynecology, Orthopedics, Pediatrics, Psychiatry, Radiology, Surgery	21	Allow students who are certain of their career choice to graduate early. Reduce student debt. Enhanced opportunity for institutional specialty mentorship.
Rutgers New Jersey Medical School	2019	Y	Internal Medicine, Pediatrics, Combined Medicine/Pediatrics	4	Address the national shortage of primary care physicians and the needs of the New Jersey Community of Newark. Reduce student debt.
Texas Tech University Health Sciences Center School of Medicine	2010	Y	Family Medicine, Primary Care	90	Expand the primary care workforce in Texas. Reduce student debt.
University of Arizona College of Medicine Tucson	2024	Y	Family Medicine	0	Increase the Family Medicine physicians practicing in Arizona. Reduce student debt.
University of Arkansas for Medical Sciences	2021	Y	Family Medicine, Internal Medicine, Pediatrics, Combined Medicine/Pediatrics	0	Increase the primary care workforce especially in Arkansas. Reduce student debt.
University of California Davis School of Medicine	2014	Y	Family Medicine, Internal Medicine-Primary Care	40	Increase the primary care workforce to practice in underserved areas. Reduce student debt.
University of Louisville Trover Campus	2011	N	Family Medicine	5	Increase the Family Medicine workforce to practice in rural areas. Reduce student debt.
University of Massachusetts Chan Medical School	2023	Y	Family Medicine, Internal Medicine, Pediatrics	0	Support individualized progression of diverse learners across the UME-GME continuum and amplify the generalist physician workforce to better meet the needs of the commonwealth and our healthcare system. Reduce student debt.
University of North Carolina	2015	Y	Family medicine, Medicine, Combined Medicine/Pediatrics, Pediatrics, Psychiatry, Surgery	19	Increase the workforce to practice in rural/underserved settings in North Carolina. Reduce student debt.
University of Pittsburgh	2023	Y	Family medicine, Internal Medicine, Pediatrics	0	Attract students to pursue primary care specialties with a goal to practice in Western Pennsylvania. Reduce student debt.
University of South Carolina School of Medicine Greenville	2024	Y	Family Medicine	0	Reduce the shortage of primary care physicians in the state of South Carolina. Reduce student debt.
University of Tennessee Health Science Center College of Medicine (UTHSC-COM)	2021	Y	Family Medicine, Internal Medicine, Medicine-Pediatrics, Neurology, Pediatrics, Psychiatry	0	Allow students who are certain of their career choice to graduate early. Reduce student debt. Address the physician workforce shortage in Tennessee.
Virginia Commonwealth University (VCU)	2017	Y	Anesthesiology, Emergency Medicine, Family Medicine, Internal Medicine, Obstetrics and gynecology, Pathology, Pediatrics, Psychiatry	14	Address the physician workforce shortage in Virginia. Reduce student debt.
Wayne State University School of Medicine	2025	Y	Anesthesiology, Dermatology Family medicine, Internal medicine, Otolaryngology, Oral Maxillofacial Surgery, Preventive medicine	0	Allows those with previous healthcare experience to progress and graduate earlier. Reduce student debt.
West Virginia University School of Medicine	2019	Y	Emergency Medicine, Family Medicine, Internal Medicine, Neurology, Obstetrics and Gynecology, Combined Medicine/Pediatrics, Pathology, Pediatrics, Psychiatry	10	Allows those who already are certain of their career choice to graduate earlier. Reduce student debt.

Of the 29 responding programs, 19 have graduates with a total of 817 graduates among all 3YMD programs. The number of graduates vary among schools with an average of 43 graduates per program and a median of 21 graduates per program. Twenty six of the 29 programs had a directed pathway to residency indicating a placement into a GME (Graduate Medical Education/Residency) program upon meeting all program objectives. The three programs without a directed pathway/GME affiliation have all of their students enter and interview in the traditional residency selection process.

Table 2 demonstrates curricular highlights for each of the programs. Most schools include an opportunity for a directed pathway experience for students, where a student completes the MD degree in three-years and then has entry into an affiliated residency program, if the student desires, linking Undergraduate and Graduate Medical Education. In addition, most of the schools report a mission to build a workforce for a specialty, for a population of patients, or geographical distribution. All schools report a mission to reduce medical student debt. The programs all meet the 130-week requirement by the LCME to confer an MD degree. Of the responding programs, the range for the total curricular weeks was 130–150 weeks with a mean of 135 weeks. The programs vary in the length of pre-clinical weeks with a range of 43–96 weeks and a median of 70 weeks.

**Table 2.** Curricular descriptions of three-year accelerated programs.

School	Number of instructional weeks in accelerated pathway	Number of weeks in pre-clerkship curriculum	Points of matriculation	USMLE Step 1 timing	USMLE Step 2 timing
Cooper Medical School of Rowan University	133	85	MS1 (beginning or end)	Before core clerkships	MS3 year after core clerkships
Geisinger Commonwealth	136	64	At matriculation	Before core clerkships	January MS3
Hackensack Meridian School of Medicine	131	64	MS3	After core clerkships	January to March MS3
James H. Quillen College of Medicine- East Tennessee State University	131	65	At matriculation, MS1	Before core clerkships	February or March MS3
Medical College of Georgia	135	68	MS1	Before core clerkships	October through December MS3
Medical College of Wisconsin- Central	137	80	At matriculation	Midway through core clerkships	June through August MS3
Medical College of Wisconsin – Green Bay	137	80	At matriculation	Midway through core clerkships	June through August MS3
Mercer University School of Medicine	134	84	MS1	Before core clerkships	February or March MS3
Medical University of South Carolina	134	67	MS2	Before core clerkships	February MS3
New York University Grossman	130	43	At matriculation, end of MS1 or MS2	After core clerkships	Summer after MS2
New York University Grossman Long Island	133	46	At matriculation	After core clerkships, July MS3	After core clerkships, August MS3
Norton College of Medicine, SUNY Upstate Medical University	130	72	At matriculation, MS1	Before core clerkships	February MS3
Ohio State University College of Medicine	136	64	At matriculation	Before core clerkships	January MS3
Penn State College of Medicine	135	65	At matriculation, MS1	After core clerkships	February MS3
Renaissance School of Medicine Stony Brook University	135	78	At matriculation, MS1	After core clerkships	March MS3
Rutgers New Jersey Medical School	151	96	At matriculation	Before core clerkships	February MS3
Texas Tech University Health Sciences Center School of Medicine	138	77	At matriculation, MS1	Before core clerkships	March, April or May MS3
University of Arizona College of Medicine Tucson	135	69	At matriculation	Before core clerkships	December or January MS3
University of Arkansas for Medical Sciences	130	71	At matriculation	Before core clerkships	Fall MS3
University of California Davis School of Medicine	132	84	At matriculation	Before core clerkships	January MS3
University of Louisville Trover Campus	130	82	MS2	Before core clerkships	December to April MS3
University of Massachusetts Chan Medical School	130	68	MS1	Before core clerkships	December MS3
University of Pittsburgh	140	67	At matriculation	January MS2	December MS3
University of North Carolina	134	67	MS1	Before core clerkships	December MS3
University of South Carolina School of Medicine Greenville	145	76	At matriculation	February or March MS2	January MS3
University of Tennessee Health Science Center College of Medicine (UTHSC-COM)	132	64	At matriculation, MS1	Before or within 6 months of starting core clerkships	December or January MS3
Virginia Commonwealth University (VCU)	131	66	MS2	Before core clerkships	December MS3
Wayne State University School of Medicine	150	72	At matriculation	Before core clerkships	April MS3
West Virginia University School of Medicine	134	78	MS2	Before core clerkships	May or June MS3

**Figure 1.**
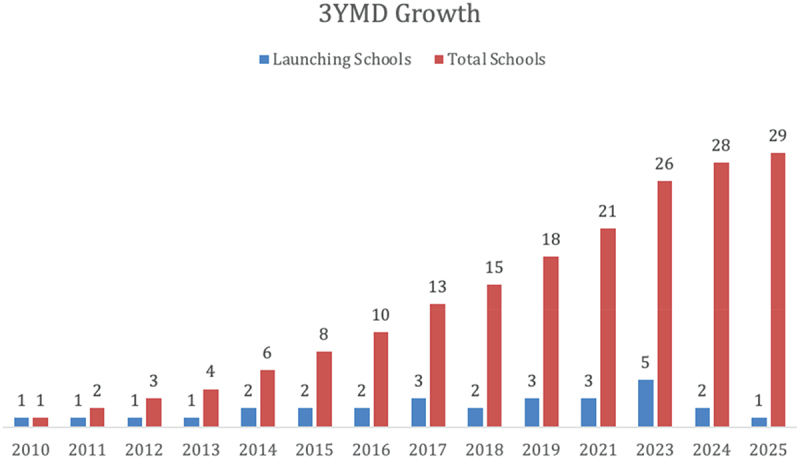
Growth of accelerated three-year programs over the last decade.

Figure 1 demonstrates the growth of Accelerated Medical Pathway Programs over time. At the time of this study, almost 20% of United States Allopathic Medical Schools have or are developing an accelerated program. Brief descriptions of the accelerated three-year MD programs are noted below.

## Discussion

Accelerated three-year medical pathway programs have grown significantly over the last decade, consistent with an overall effort to redesign medical curricula, and make medical education more efficient and affordable. The accelerated pathway is commonly established as an alternative pathway within a school’s traditional medical degree program, but with a few notable examples, the pathway rarely comprises a campus of a medical school or an entire school.

While the structure of each 3YMD program is unique to the medical school based on the unique underlying curriculum, several common tenets are present. Programs significantly decrease/remove vacations or unstructured curricular time. The traditional ‘vacation’ between the first and second year of medical school is replaced with curricular activities. Programs also utilize longitudinal clinical experiences to increase the curricular footprint throughout the three years.

While many accelerated programs initially focused on developing a primary care workforce, many recent or expanded programs also center on providing students interested in a broad range of specialties earlier exposure and access to residency training. In all cases, accelerated pathways are designed for students who have identified a career pathway early and have shown commitment to that specialty, allowing them to forgo some of the experiences or electives associated with career exploration. Almost all reported programs (26/29) have a directed UME to GME pathway within the medical school’s associated residency programs. A directed pathway indicates a placement to match into the residency program, assuming completion of the program objectives. Students are permitted to apply to other programs, should they choose to do so. In most cases, students in their final year of medical school (usually the third year), are required to enter into the National Resident Matching Program (NRMP). The NRMP has an all-in exemption policy that permits placement outside without participation in the match. Only 3 of the 29 programs have the exemption, the remainder are able to achieve the intended outcomes of the acceleration and directed pathway without the exemption. Moreover, students in good academic standing in the accelerated pathway who choose to match at the residency program linked to their medical school/healthcare system, will successfully match into that program. A handful of programs in CAMPP have an All-In Policy Exception from the NRMP, and those accelerated students do not participate in the open Match. Rather, in these programs, the complement of the program is decreased to accommodate the accelerated students and the process automatically places the students in that program. Across all members of the CAMPP, member schools’ accelerated curricula smooth the transition from UME to GME and facilitate targeted workforce development.

As a consortium of schools that have developed, implemented, and continue to refine innovation in medical education via three-year pathways, the CAMPP members have facilitated collaboration, sharing of best practices, and navigating regulatory requirements. Shared data and experiences among member schools offer a wide range of opportunities for program evaluation and educational research. Early outcomes indicate students are equally prepared and graduate with less debt [7,8]. In a time-fixed structure of medical education, the accelerated curricula allow for some time-variability for students who know their future specialty and are prepared for graduation. It also adds to the discussion around a transition to a competency-based medical education (CBME) framework, that students can achieve needed competencies for graduation in less than the traditional four-year structure. Since most programs are small and new, one long-term goal of the consortium is to study the aggregated cohort of accelerated students in a longitudinal manner as they progress through residency and enter into practice. Although the current CAMPP members are allopathic medical schools, the concept of an accelerated pathway is likely transferable to osteopathic medical schools given the curricula are tailored to meet the needs of the school and workforce. Future research goals include assessment of long-term impact of acceleration on student debt, physician workforce, wellness and job satisfaction, professional identity formation and other issues related to allowing physicians to enter the workforce earlier.

## Supplementary Material

CAMPP_Institution_survey.docx
