# Supplementary material for: Accelerated 3YMD programs: the last decade of growth of the Consortium of Accelerated Medical Pathway Programs (CAMPP)
Source: Med Educ Online. 2024 Sep 27;29(1):2400394. doi: 10.1080/10872981.2024.2400394 (PMC11441013; doi:10.1080/10872981.2024.2400394)
Supplement: CAMPP_Institution_survey.docx [file ZMEO_A_2400394_SM2303.docx]

CAMPP Institution Update

Start of Block: Default Question Block

Q1 Institution

________________________________________________________________

Q2 Year Founded OR Anticipated Start Year

________________________________________________________________

Q3 Please describe the Mission of your accelerated program.

Q4 Through the graduating class of 2023 -
How many students TOTAL have matriculated into your program?

________________________________________________________________

Q5 Through the graduating class of 2023 -
How many of your students completed your program on schedule?

________________________________________________________________

________________________________________________________________

Q6 Total number of students per class at your institution (4 year program)

________________________________________________________________

Q7 Number of instructional weeks of the accelerated program (or anticipated)

________________________________________________________________

Q8 Preclinical curriculum length

________________________________________________________________

Q9 Specialty options offered

________________________________________________________________

________________________________________________________________

Q10 Admissions Process (select all that apply)

- Admission on matriculation (1)
- Admission during M1 year (2)
- Admission during M2 year (3)
- Other (Specify) (4) __________________________________________________

Q11 USMLE Step 1 Scheduling Time frame Before or after Clerkship

- Before Clerkship (4)
- After Clerkship (5)
- Other (Specify) (6) __________________________________________________

Q12 USMLE Step 2 Scheduling Time frame (Month and School Year i.e. November MS3 year)

________________________________________________________________

Q13 Please select program features (select all that apply)

- Longitudinal Clerkship/curriculum: (1)
- Match Waiver (Y/N/In process): (2)
- Match Process (Internal/External/Both/Other): (3)
- Post-Residency Service requirement: (4)
- Other Notable Features (5) __________________________________________________
